# A system-wide investigation into the phosphoregulatory network of TNIK and its cellular implications

**DOI:** 10.3389/fbinf.2026.1722876

**Published:** 2026-03-13

**Authors:** Akhila Sheela, Suhail Subair, Samseera Ummar, Althaf Mahin, Athira Perunelly Gopalakrishnan, Rajesh Raju, Sowmya Soman

**Affiliations:** Centre for Integrative Omics Data Science (CIODS), Yenepoya (Deemed to be University), Mangalore, Karnataka, India

**Keywords:** carcinogenesis, cell migration, co-regulation, phosphoproteomics, TNIK

## Abstract

**Introduction:**

TNIK (Traf2- and Nck-interacting kinase) is a serine/threonine kinase that plays a crucial role in cytoskeletal organization, Wnt pathway activation, and cancer progression. Recent studies have implicated the role of TNIK in oncogenic signaling pathways and neuropsychiatric regulation. However, the phosphosignaling dynamics of TNIK remain largely unknown.

**Methods:**

To explore TNIK phosphoregulation, we systematically assembled and integrated global human phosphoproteomic datasets. We identified the predominant phosphosites based on the frequency. Relative solvent accessibility (RSA) and Phosphosite accessibility index (PAI) were calculated to determine the solvent exposure and structural flexibility of TNIK predominant phosphosites. To assess the functional significance of TNIK, we examined proteins that were differentially co-regulated with its predominant phosphosite, along with the corresponding upstream kinases, downstream substrates, and interacting proteins.

**Results:**

Analysis of the global human cellular phosphoproteome datasets revealed phosphosites S640, S680, S707, and S769 of TNIK to be the most frequently perturbed phosphosites across diverse experimental conditions. The results of the RSA and PAI analysis revealed that the predominant sites are located within highly solvent-exposed and structurally flexible regions. Notably, we obtained a large number of co-regulated proteins that were associated with cell growth, carcinogenesis, and apoptosis. The interactors identified were primarily enriched towards carcinogenesis. Our analysis revealed PRKAA1 and RPS6KB2 as robust upstream kinases of TNIK_S640 and TNIK_S707. We also identified many proteins involved in RNA splicing, cytoskeletal organisation, and cell migration as potential downstream substrates of TNIK.

**Discussion:**

Considering the challenges in targeted experimental analysis of these sites, a global co-regulation analysis approach was employed. Our results show that these phosphorylation sites in TNIK can influence carcinogenesis and related biological functions. It offers new insights into TNIK-mediated cellular functions, deepening our comprehension of its involvement in carcinogenesis and RNA splicing.

## 1 Introduction

Traf2-and Nck-interacting kinase (TNIK) is a serine/threonine kinase, a member of the germinal center kinase (GCK) family which belongs to the Ste20 group of kinases (Fu et al., 1999). The cDNA of TNIK was initially cloned from the human brain and reported as the first GCK member potentially involved in cytoskeletal regulation (Fu et al., 1999). It is encoded by the *TNIK* gene, located on chromosome 3q26.2-q26.3. The canonical TNIK protein consists of 1360 amino acids (154,943 Da) and has eight alternatively spliced isoforms of varying lengths. It is highly expressed in the heart, brain, and skeletal muscles and is located primarily in the cytoplasm, cytoskeleton,recycling endosome, and nucleus (Fu et al., 1999). TNIK is a multidomain protein, consisting of an N-terminal kinase domain, a central intermediate domain, and a C-terminal citron homology (CNH) domain. The intermediate domain is responsible for binding to adaptor proteins such as TRAF2 and NCK (Kukimoto-Niino et al., 2022). Functionally, TNIK plays a critical role in actin cytoskeleton regulation (Taira et al., 2004), cytoskeleton organization (Fu et al., 1999), microvillus assembly (Gloerich et al., 2012), regulation of dendrite morphogenesis (Kawabe et al., 2010), positive regulation of JNK cascade (Taira et al., 2004), and conserved regulation of glucose and lipid metabolism in obesity (Pham et al., 2023). It regulates the Wnt signaling pathway by activating its target genes (Mahmoudi et al., 2009), (Raju et al., 2011). Furthermore, TNIK is implicated in many cancers, such as ovarian cancer (Wu et al., 2024), colorectal cancer (Hu et al., 2023; Zhou et al., 2023), (Takahashi et al., 2020), thyroid cancer (Li et al., 2023), lung squamous cell carcinoma (Torres-et al., 2021), and osteosarcoma (Hirozane et al., 2021). Moreover, a study demonstrates that knockdown of TNIK leads to increased apoptosis and reduced cell viability in lung squamous cell carcinoma (LSCC) (Torres-et al., 2021). Additionally, TNIK mutations are a genetic cause of intellectual disability in the context of MRT54 (Anazi et al., 2016).

TNIK phosphorylation is crucial for its role in pathways like Wnt signaling, MAPK signaling, and cytoskeletal organization. TNIK promotes castration-resistant prostate cancer (CRPC) progression by directly interacting with EGFR and phosphorylating it, which enhances its nuclear import and transcriptional activation, leading to increased EGFR signaling (Guo et al., 2024). TNIK also plays a role in the MAPK signaling pathway, affecting ERK1/2, p 38, and JNK1-3 (Jiang et al., 2024). TNIK can phosphorylate and regulate proteins involved in actin regulation, synaptic adhesion, and synaptic plasticity, suggesting a role in cytoskeletal organization (Fu et al., 1999). In addition to that, phosphorylation of TNIK at S764 is reported to be associated with prostate cancer (Lee et al., 2019). TNIK activates JNK signaling through its GCKH domain independent of kinase activity and potentially modulates cytoskeletal organization (Fu et al., 1999).

At the molecular level, we identified enriched phosphorylation in the intermediate region between the kinase domain and CNH domain of TNIK. The relevance of phosphorylation in this region or their role in the regulation of TNIK functions remains underexplored. Further, the crystal structure covering the entire protein is currently unavailable, making it difficult to interpret the structural alterations. This lack of molecular details on TNIK hinders our ability to comprehend the variations in signaling dynamics that contribute to disease associations. In the current study, we investigated the phospho-signaling dynamics associated with these regions to interpret the molecular-level details.

## 2 Materials and methods

### 2.1 Screening and identification of TNIK predominant phosphorylation sites

An extensive PubMed search was conducted to identify high-confidence TNIK phosphosites by screening global phosphoproteomics datasets from human cells and tissues, focusing on Class 1 phosphosites, which have an A-score ≥13 and localization probability ≥75%. The A-score provides a measure of confidence in phosphorylation site localization based on the presence and intensity of site-determining ions in MS/MS spectra, localization probability reflects the likelihood of correct site placement. The analysis was conducted based on the methodologies that had been previously established in our laboratory (Priyanka et al., 2024), (Raghu et al., 2025).

Datasets were classified into qualitative profile datasets, where test conditions and controls were considered independent phosphosite profiles, and quantitative differential datasets, where phosphosite abundance was compared between test biological/experimental conditions and their corresponding controls. In quantitative datasets, phosphosites were considered upregulated if the fold change was ≥1.3, and downregulated if ≤0.76, with a p-value <0.05. All phosphosites were mapped to HGNC gene symbols (as of 30.05.2023) and UniProt accessions (Priyanka et al., 2024; UniProt Consortium, 2023) using our in-house mapping tool. For effective categorisation, the annotated biological and experimental conditions were attached to every dataset in a standard format (Mahin et al., 2025). Class 1 phosphosites obtained from the profiling and differential expression datasets were further analysed for the detection of predominant TNIK phosphosites. Each phosphosite was considered predominant if it was found in more than 50% of profiling and differential expression datasets. Phosphosites identified using specific phospho-antibodies or mutation-based approaches but not frequently reported as class-1 sites in these datasets, are currently not accounted for as part of this analysis.

### 2.2 Sequence conservation analysis and phylogenetic mapping of predominant TNIK phosphosites

We retrieved homologous TNIK protein sequences using BLASTp (Basic Local Alignment Search Tool) against the UniProtKB reference proteome, with the human TNIK sequence (UniProt Q9UKE5) as the query. Top BLAST hits spanning major metazoan taxa (from invertebrates to mammals) were collected to represent TNIK orthologues. These sequences were aligned with Clustal Omega (a scalable MSA program), yielding a multiple sequence alignment covering all conserved domains of TNIK. We then computed Shannon entropy (H) for each alignment column as a measure of sequence variability. Entropy was calculated as

$$H=-\sum_{i}p_{i}\log_{2}\left(p_{i}\right)$$

Where pi is the frequency of amino acid i at that position. Lower H implies higher conservation. For interpretability, we defined thresholds: H < 0.10 as highly conserved, 0.10 ≤ H ≤ 0.50 as moderately conserved, and H > 0.50 as poorly conserved. Finally, to infer the ancestral origin of each phosphosite, we examined the taxonomy of BLAST hits. The broadest taxonomic clade encompassing all significant orthologues was taken as the site’s phylogenetic origin. In practice, this involved mapping each hit’s species to the NCBI taxonomy and identifying the lowest common ancestor node (e.g., Bilateria, Tetrapoda, *etc.*) for the site (Liang et al., 2023; Ochoa et al., 2020) (Priyanka et al., 2024).

### 2.3 Relative solvent accessibility (RSA) and phosphosite accessibility index (PAI)

As the functional phosphosites are confined predominantly in the flexible and solvent accessible regions, we performed relative solvent accessibility (RSA) and phosphosite accessibility index (PAI) analysis of the predominant sites of TNIK. RSA and PAI were computed using the DSSP algorithm applied to the AlphaFold structural model using Biopython implementation (DSSP version 4.2.2). For each phosphorylation site, RSA values were normalized to a 0–1 scale, where higher values indicate increased solvent exposure. Residues assigned to coil or loop regions were classified as structurally flexible and assigned a loop indicator value of 1, while residues within α-helices or β-sheets were assigned a value of 0. AlphaFold pLDDT (predicted Local Distance Difference Test) scores were extracted directly from the B-factor column of the PDB file and normalized by division by 100 to obtain values between 0 and 1, representing local structural confidence. To quantitatively integrate multiple structural descriptors into a single interpretable metric, a Phosphosite Accessibility Index (PAI) was defined as:

PAI=0.5×RSAnorm+0.3×pLDDTnorm+0.2×Loop

This weighting scheme prioritizes solvent accessibility while incorporating structural confidence and flexibility, capturing the key determinants of phosphorylation accessibility potential.

### 2.4 Identification of phosphosites on other proteins co-regulated with the predominant TNIK phosphosites

In order to identify phosphosites in other proteins (PsOPs) that are showing expression-coregulation with predominant phosphosites of TNIK, quantitative datasets were grouped based on whether TNIK sites were upregulated (U) or downregulated (D) (Table 1). When the expression of both TNIK phosphosite and PsOPs was upregulated, it was categorized as UU. However, when TNIK phosphosite was upregulated, and PsOPs were downregulated, they were categorised as UD. In the case of downregulation of TNIK phosphosite and upregulation of PsOPs, they were classified as DU, and when PsOPs were downregulated in comparison with downregulated TNIK phosphosite, they were designated as DD. PsOPs in UU and DD (UUDD group) represent positive co-regulation, while those in UD and DU (UDDU group) represent negative co-regulation.

**TABLE 1:** Classification of TNIK-PsOP phosphosite co-regulation patterns.

TNIK phosphosite regulation	PsOP regulation	Category	Interpretation
Upregulated (U)	Upregulated (U)	UU	Positive co-regulation
Upregulated (U)	Downregulated (D)	UD	Negative co-regulation
Downregulated (D)	Downregulated (D)	DD	Positive co-regulation
Downregulated (D)	Upregulated (U)	DU	Negative co-regulation

We counted the number of datasets supporting each PsOP–TNIK relationship and applied one-sided Fisher’s exact tests (FET) to evaluate statistical significance (Priyanka et al., 2024). High-confidence TNIK phosphosite-PsOP pairs were defined as those that met four main criteria with a differential FET p-value <0.05, a ratio meeting at least 10% of total differential datasets, and presence in at least three distinct studies and three unique experimental conditions.

### 2.5 Analysis of co-occurrence sites within TNIK

A co-occurrence analysis was carried out to look at the mutual associations of phosphorylation sites within TNIK by examining the co-differential regulation pattern of phosphosite pairs within TNIK. Multiple TNIK phosphosites found under the same experimental conditions were extracted from each differential data set. We determined the UU, UD, DD, and DU frequencies for each pair independently. We then used ∑ (nUU + nDD)/∑ (nUD + nDU) to evaluate the positive co-regulation frequency and ∑ (nUD + nDU)/∑ (nUU + nDD) to evaluate the negative co-regulation frequency. The differential co-regulation frequency of the phosphosite pair is listed in Supplementary Table S1.

### 2.6 Identification of interactors, upstream kinases, and downstream substrates of TNIK

The protein interactors of TNIK were gathered from various databases based on experimental characterisation. These comprised Spike (Paz et al., 2011), BioGRID (Oughtred et al., 2021), and IntAct (Orc et al., 2014). The kinases and downstream substrates of specific TNIK phosphosites were predicted by multiple tools, such as NetworKIN (downloaded on 04.01.2023) (Linding et al., 2008) and AKID (downloaded on 24.05.2023) (Parca et al., 2019). Significantly, we included TNIK phosphosite specific kinases based on the synthetic peptide screening study conducted by Johnson et al. for the assessment of the substrate specificity of the human kinome, with a cutoff of 90 percentile (Johnson et al., 2023).

### 2.7 Data visualisation

The R/Bioconductor package trackViewer (10.18129/B9. bioc.trackViewer) (Ou and Zhu, 2019) is utilized for the representation of data through lollipop plots. Cytoscape (Shannon et al., 2003) is used for visualising the interaction.

## 3 Results

### 3.1 TNIK phosphosite map from the global phosphoproteome

**FIGURE 1:**
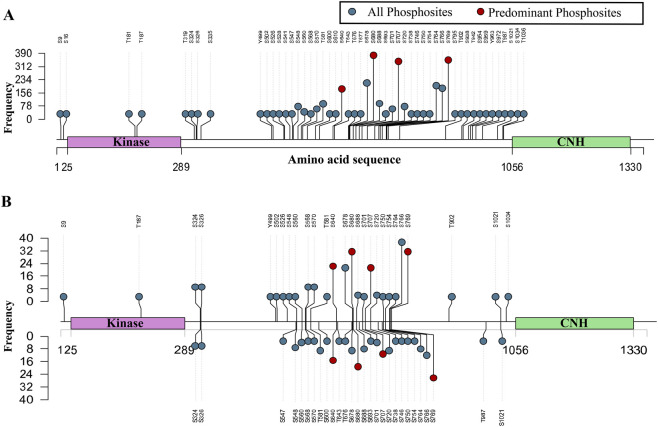
Lollipop plot visualization of phosphosites within TNIK with their detection frequencies in global cellular phosphoproteomics datasets. The *x*-axis represents the TNIK protein sequence, with annotated functional domains including the protein kinase domain (Kinase) and the citron homology (CNH) domain. The *y*-axis indicates the frequency of phosphosite detection across datasets **(A)** Class-1 phosphosites detected in TNIK across 673 global cellular qualitative profile datasets. **(B)** Class-1 phosphosites detected in TNIK across 182 global quantitative differential profile datasets (upward-oriented lollipops represent the frequency of phosphosite upregulation, whereas downward-oriented lollipops represent the frequency of phosphosite downregulation.

To analyze the functionally significant phospho-signaling patterns associated with TNIK, we screened over 3825 publicly available human global cellular phosphoproteomics datasets and identified 182 quantitative differential datasets and 673 qualitative profiling datasets containing class-1 TNIK phosphosites. Systematic mapping of phosphosites within TNIK revealed 51 phosphosites from profiling datasets (Figure 1A) and 35 phosphosites with differential expression data (Figure 1B). The datasets and the information on the qualitative and quantitative datasets are provided in Supplementary Table S2.

Among the 35 phosphosites within differential data, four phosphosites, S640, S680, S707, and S769, showed the highest differential expression frequencies (Wang et al., 2014; Balan et al., 2006; Rena et al., 2003; Rena et al., 2003; Walle et al., 2015). At the sequence level, these phosphosites were within the heavily phosphorylated region of TNIK (amino acid range: 289–1056). Further analysis was carried out on these phosphosites to interpret their phosphoregulatory patterns and functional significance.

### 3.2 Evolutionary origin and conservation of TNIK phosphosites

The site-specific shannon entropy analysis yielded the following conservation scores: S640 H = 0.074 (highly conserved), S680 H = 0.517 (low conservation), S707 H = 0.126 (moderate), and S769 H = 0.141 (moderate) (Supplementary Table S3). Based on our thresholds, S640 is classified as highly conserved, both S707 and S769 as moderately conserved, and S680 as poorly conserved. Mapping these sites with its 3 residue context to phylogenetic origin gave: S640 in the Boreoeutheria (placental mammal) clade (estimated ∼90–100 million years ago), S680 in the Bilateria (the common ancestor of all bilaterian animals, ∼800+ Mya), and both S707 and S769 in Euteleostomi (bony vertebrates, ∼435 Mya). Thus, the oldest site (S680, bilaterian origin) shows the highest entropy (least conserved), whereas the youngest site (S640, Boreoeutherian origin) is the most conserved. The two intermediate-age sites (S707/S769, euteleostome origin) have intermediate entropy.

### 3.3 Solvent exposure and structural flexibility of TNIK predominant sites

Mapping of experimentally observed TNIK predominant sites onto the AlphaFold structural model revealed that all analyzed serine residues (S640, S680, S707, and S769) are located within highly solvent-exposed and structurally flexible regions (Supplementary Table S4). All four sites were classified as loop regions, consistent with increased accessibility to upstream kinases.

Among these residues, S769 exhibited the highest structural accessibility, with an RSA of 0.992 and a PAI score of 0.809, indicating near-complete solvent exposure despite moderate local structural confidence (pLDDT = 37.75).

Residues S707 and S640 also demonstrated high accessibility, with RSA values of 0.862 and PAI scores of 0.728 and 0.723, respectively. These sites occupy extended loop regions with substantial solvent exposure, supporting their recurrent detection in phosphoproteomic datasets. Residue S680, while slightly less exposed (RSA = 0.823), still displayed a high PAI score (0.705).

### 3.4 Co-regulated phosphorylation dynamics associated with TNIK predominant phosphosites

A co-regulation analysis approach was employed to investigate the phospho-signaling patterns associated with the predominant TNIK phosphosites. In this context, we examined the co-expression patterns of phosphosites in other proteins (PsOPs), which tend to show expression co-regulation with the predominant phosphosites of TNIK consistently. To accomplish this, we implemented strict inclusion and exclusion criteria, including a ≥10% frequency cutoff from the total differential frequency of the predominant site for positive or negative co-regulation, Fisher’s Exact Test (FET) *p*-value <0.05, correlation based on data from at least three publications (PMID confidence), and regulation across different experimental conditions (code count) in a minimum of three distinct conditions. After applying stringent inclusion-exclusion criteria for quality control, we observed 1349 PsOPs positively co-regulated with TNIK_S640 and 77 PsOPs negatively co-regulated with TNIK_S640. Similarly, TNIK_S680 showed 729 positively co-regulated and 75 negatively co-regulated PsOPs, while site S707 had 356 positively co-regulated and 247 negatively co-regulated phosphosites. Additionally, phosphosite S769 showed 650 positively co-regulated and 94 negatively co-regulated phosphosites within the high-confidence co-regulation criteria (Supplementary Table S5).

PEA15_S116, SMC4_S28, HTT_S1874, NMD3_S468, ZC3H13_S265, DDX59_S160, PNKP_S114, and CBX8_S256 were among the proteins showing high positive co-regulation with TNIK_S640. PEA15_S116 was the top positively co-regulated phosphosite, whereas PLEKHA5_S855 showed the top negative co-regulation with TNIK_S640. PEA15 is a modulator of signaling pathways that control apoptosis and cell proliferation (Rena et al., 2003), with phosphosite S116 reported to induce carcinogenesis (Wu et al., 2021) and inhibit apoptosis (Eckert et al., 2008). Meanwhile, PLEKHA5 is a novel downstream effector of the oncogene MET and a critical regulator of malignant phenotypes (Nagamura et al., 2021). Similarly, FXR2_S603, HIRIP3_S159, TRIM24_S1025, and HIRIP3_S160 were among the proteins showing high positive co-regulation with TNIK_S680. FXR2_S603 was the top positively co-regulated phosphosite, whereas PPIP5K2_S493 showed top negative co-regulation. FXR2 is an RNA-binding protein that regulates neurogenesis (Guo et al., 2011) while PPIP5K2 is one of the PPIP5K isoforms responsible for the synthesis of diphosphoinositol polyphosphates (Wang et al., 2014). BPTF_S572, ABI1_S231, RIOK2_S335, LMNA_S66, and PSIP1_S177 were among the proteins showing high positive co-regulation with TNIK_S707. BPTF_S572 was the top positively co-regulated phosphosite, whereas ANLN_S323 showed top negative co-regulation. BPTF is a core subunit of the nucleosome-remodeling factor (NURF) complex, and plays an essential role in chromatin remodeling (Zhao et al., 2019), while ANLN is involved in cytokinesis and cytoskeletal remodeling (Wang et al., 2025). SRSF2_S212, SIRT1_S47, LRRFIP2_S328, PHRF1_S1371, and TRIM24_S1028 were among the proteins showing high positive co-regulation with S769 of TNIK, SRSF2_S212 was the top positively co-regulated phosphosite, whereas WASHC2A_S288 showed top negative co-regulation. SRSF2 is a core regulator of RNA splicing (Ma et al., 2023), while WASHC2A regulates Arp2/3 complex-mediated actin nucleation (Cerdán-Vélez and Tress, 2023). However, the direct association of these proteins with TNIK is currently unknown. In addition, the molecular significance of these phosphorylations to these proteins is also currently unknown.

### 3.5 Co-occurrence analysis of TNIK phosphosites

**FIGURE 2:**
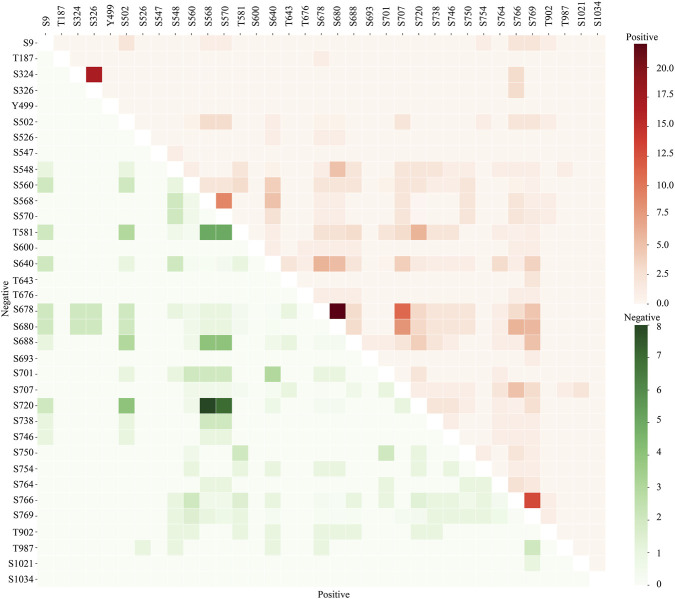
Co-occurring phosphosite pairs within TNIK. Heatmap representing the co-occurrence pattern of phosphosites with TNIK. The upper red triangle indicates positive expression co-regulation, and the lower green triangle indicates negative expression co-regulation between phosphosite pairs of TNIK, with colour gradient representing the frequency of co-occurrence.

The co-occurrence of phosphorylation sites is often associated with functional similarity and evolutionary conservation (Li et al., 2017). To explore this in the context of TNIK, we examined the co-occurrence of differential expression of its predominant phosphosites S640, S680, S707, and S769 within the heavily phosphorylated region. Interestingly, these phosphosites from four different peptides exhibit a moderate co-occurrence frequency between them. Further, expanding our analysis toward the co-regulation pattern of phosphosite pairs within differential data, we noted that among the co-occurred phosphosite pairs, the S640 showed a higher co-occurrence frequency with the other predominant phosphosites. The phosphosite pairs S640 and S680 showed positive co-occurrence with a frequency of 5.3, S640 and S769 with a frequency of 5.6, and S640 and S707 with a frequency of 4 (Figure 2).

Although the co-regulation frequency is low, phosphosites of 155 proteins were positively co-regulated with their phosphosites were positively co-regulated together with S640 and S680. Similarly, 50 were positively co-regulated together with S640 and S707, and 87 proteins with their phosphosites were positively co-regulated with S640 and S769. Among them, the predicted substrates KIF2A_T799, RBM15_S292, EIF4EBP1_T10, HNRNPM_S618 and VIM_S51 showed positive co-regulation with S640 and S680. JPH2_T490, SRF6_S297, ZNF608_T636 and SRMM2_S2067 showed positive co-phosphoregulation with S640 and S769. KIF4A is a kinesin-4 family member that plays an important role in regulating mitosis (Dong et al., 2018), with its T799 site associated with its induced microtubule-dependent ATPase activity of KIF4A and cytoskeletal reorganization (Dong et al., 2018; Nunes Bastos et al., 2013). VIM is an intermediate filament protein that plays a role in cell processes, including cell migration and cell shape (Arrindell and Desnues, 2023), with its phosphosite S57 associated with cytoskeletal reorganization (Goto et al., 2002). Together, this indicates that the phosphoregulation in these 130 aa regions significantly contributes to the TNIK kinase-dependent and/or independent functions.

### 3.6 Co-phosphoregulation between TNIK and binary interactor phosphosites

To analyze co-phosphoregulated phosphosites in proteins directly associated with TNIK, we compiled a list of its binary interactors from publicly available databases, including BioGRID, IntAct, and Spike (Supplementary Table S6). A total of 299 binary interactors were identified for TNIK. Our analysis revealed 59 interactors with their phosphosites positive expression co-regulation with S640, while four showed negative co-regulation. Similarly, 38 PsOPs were positively co-regulated with S680, while 3 showed negative co-regulation. Additionally, 14 PsOPs were positively co-regulated with S707, while 2 were negatively co-regulated, and 17 PsOPs were positively co-regulated with S769, while four were negatively co-regulated. Among them, we found phosphosite expression co-regulations of TNIK with 3 phosphosites in NCOR2, where S1775 positively co-regulated with TNIK_S640 and S1018 with S680, while S1479 negatively co-regulated with S640. Similarly, 2 phosphosites in DPYSL3 showed positive expression co-regulation with TNIK. DPYSL3_T509 showed positive co-regulation with S640, S680, and S707 of TNIK. The site T509 is associated with induced carcinogenesis (Braekeveldt et al., 2018), while DPYSL3_S522 is showing positive co-regulation with S640 of TNIK, where S522 is associated with altered cell motility (Cole et al., 2006). RAF1 kinase, the signaling molecule functioning in the Ras pathway to transmit mitogenic, differentiative, and oncogenic signals, is also showing positive expression co-regulation with TNIK sites. RAF1_S301 is showing positive expression co-regulation with TNIK_S680, which is also associated with induced cell differentiation (Rashid et al., 2020) and induced kinase activity (Balan et al., 2006), while RAF1_S296 is positively co-regulated with TNIK_S769. GSK3A_S14 is showing positive expression co-regulation with TNIK_S680, while S21 positively co-regulates with TNIK_S707. The S21 site has been reported to be associated with carcinogenesis (Liu et al., 2023) and apoptosis (Gao et al., 2015). Additionally, most of the co-regulation patterns of protein phosphosites are associated with cytoskeletal reorganization, induced carcinogenesis, cell motility, and apoptosis (Table 2).

**TABLE 2:** Phosphosites in binary interactors that are positively co-regulated with TNIK predominant sites.

TNIK phosphosite	Co-regulated protein phosphosite	Phosphosite-associated function	Reference
S640	DPYSL3_T509	Carcinogenesis	Braekeveldt et al. (2018)
S640	PTPN11_Y542	Carcinogenesis, apoptosis, cytoskeletal reorganization	Karaca et al. (2022)
S640	RANBP1_S60	Carcinogenesis, apoptosis	Huynh et al. (2019)
S640	SIRT1_S47	Carcinogenesis, apoptosis	Yao et al. (2022)
S640	RCC1_S11	Cytoskeletal reorganization	Horiike et al. (2009)
S640	SLC9A1_S703	Cell motility, cytoskeletal reorganization	Walle et al. (2015)
S640	MACF1_S7330	Cell motility, cytoskeletal reorganization	Wu et al. (2011)
S680	DPYSL3_T509	Carcinogenesis	Braekeveldt et al. (2018)
S680	MACF1_S7330	Cell motility, cytoskeletal reorganization	Wu et al. (2011)
S680	DSTN_S3	Cytoskeletal reorganization	Amano et al. (2001)
S707	DPYSL3_T509	Carcinogenesis	Braekeveldt et al. (2018)
S707	GSK3A_S21	Carcinogenesis, apoptosis	Liu et al. (2023)
S707	GSK3B_S9	Carcinogenesis	Huang et al. (2023)
S707	SET_S7	Carcinogenesis, cell motility	Yin et al. (2019)
S707	DSTN_S3	Cytoskeletal reorganization	Amano et al. (2001)
S769	SIRT1_S47	Carcinogenesis, apoptosis	Yao et al. (2022)

### 3.7 Potential upstream kinases associated with TNIK

**FIGURE 3:**
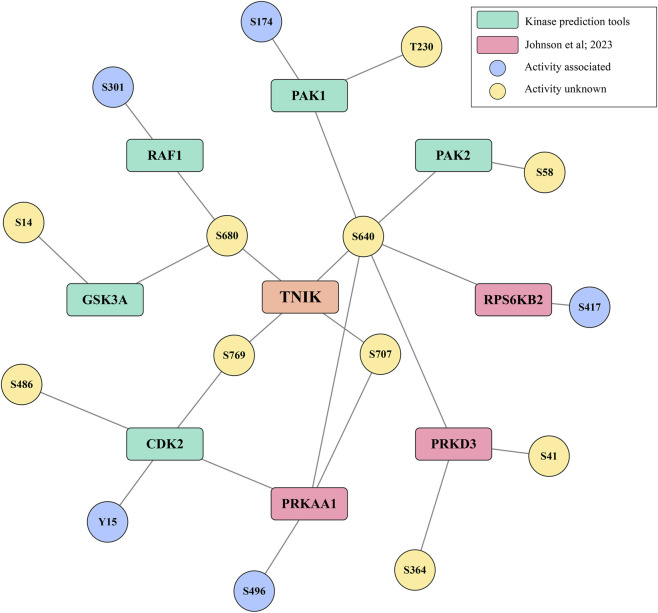
Phosphorylation status of upstream kinases associated with predominant phosphosites of TNIK. A network depicting predicted upstream kinases and their phosphosites in predicted kinases that showed positive co-phosphoregulation with respective substrate phosphosites in TNIK.

Phosphorylation is one of the most significant types of signal transduction in cells, which is solely mediated by protein kinases. Clarity on upstream kinases that mediate phosphorylations in TNIK is required to comprehend TNIK signal transduction better. Currently, no upstream kinases have been reported to phosphorylate TNIK phosphosites. In our efforts to better interpret TNIK signal transduction, we fetched the list of potential upstream kinases that can phosphorylate TNIK’s predominant sites from multiple sources. To give an added layer of confidence, we mapped the phosphosite expression co-regulations in these potential upstream kinases with the respective predominant phosphosites of TNIK (Supplementary Table S7). Our analysis identified five kinases upstream to S640, two kinases upstream to S680, one upstream to S707, and one upstream to S769 of TNIK with high-confidence phosphosite co-regulations (Figure 3).

5′-AMP-activated protein kinase (PRKAA1), which is a negative regulator of mTOR signaling, is identified as a potential upstream kinase of TNIK_S640. Our analysis revealed positive expression co-regulation of PRKAA1_S496 with TNIK_S640. The phosphorylation at site S496 is associated with its reduced AMPK activity (Heathcote et al., 2016). Similarly, RPS6KB2, which is also a downstream substrate of mTOR signaling, is identified as a potential upstream kinase of TNIK_640. RPS6KB2_S417 is associated with its induced activity and selective mRNA translation (Martin et al., 2001). Additionally, members of the p21-activated kinase (PAK) family, PAK1 and PAK2, which are also involved in a protein complex with TNIK, are identified as potential upstream kinases of TNIK. PAK2_S58, PAK1_T230, and PAK1_S174, which are known to be both activity-induced and involved in cell cycle regulation (Kong et al., 2009), exhibit positive expression co-regulation with TNIK S640. Further, positive expression co-regulation with TNIK S640 was noted with phosphosites in potential upstream kinases such as PRKD3_S364 and S417. The kinases RAF1 and GSK3A, which are associated with cellular processes like cell growth, proliferation, and apoptosis (Huang et al., 2017), (Grassilli et al., 2014), were identified as potential upstream kinases of TNIK at S680. RAF1_S301 and GSK3A_S14 are positively co-regulated with TNIK_S680. PRKAA1_S486 is showing positive expression co-regulation with TNIK_S707. Phosphorylation at Y15, an inhibitory site of CDK2 involved in cell cycle regulation (Coulonval et al., 2003), was also positively co-regulated with TNIK S769.

### 3.8 Potential downstream substrates of TNIK

Currently, four (PRKAA2, HTT, SMAD1, and TCF4) experimentally validated substrates have been mapped to be phosphorylated by TNIK. To further explore the substrates of TNIK, we fetched the list of potential downstream substrates that can be phosphorylated by TNIK predominant sites from multiple sources. To give an added layer of confidence, we mapped the phosphosite expression co-regulations in these potential downstream substrates with the respective predominant phosphosites of TNIK. Our analysis identified 43 predicted candidate downstream substrates with S640, 25 downstream with S680, 26 substrates downstream with S707, and 21 substrates downstream with S769 of TNIK with high-confidence positive phosphosite co-regulations (Supplementary Table S7).

Ribosomal protein RPS14, which is also a binary interactor of TNIK, is identified as a predicted downstream substrate of TNIK. RPS14_S139 and T140 are positively co-phosphoregulated with TNIK_S640. Similarly, BAIAP2 and CTNND1, which are the complex interactors of TNIK, are identified as potential downstream substrates of TNIK. CTNND1_T201 is showing positive expression co-regulation with TNIK_S640, while T340 of BAIAP2 is showing positive co-regulation with TNIK_S640. Phosphorylation at T340 acts as a negative regulatory switch for BAIAP2 and is associated with cytoskeletal reorganization (Robens et al., 2010). In addition, 5 proteins SRSF5, PRPF40A, SRSF6, HNRNPM, and CHERP involved in the ‘spliceosome’ pathway were identified as potential substrates for TNIK_S640. GSK3A, a binary interactor of TNIK, was identified as both potential upstream kinase and a downstream substrate of TNIK_S680. GSK3A_S14 is positively co-regulated with S680 of TNIK. Furthermore, proteins CLIP1 and CLASP1, which are reported to function in the regulation of microtubule cytoskeleton organization, were identified as potential substrates for TNIK. CLIP1 is a member of microtubule (MT) plus-end tracking proteins that regulate microtubule dynamics (Larti et al., 2015). CLIP1 S204 is showing positive expression co-regulation with TNIK_S680. CLASP1_T1095 and S594 are positively co-regulated with TNIK_S680. CLASP1 is required at kinetochores for attached microtubules to exhibit normal dynamic behavior (Maiato et al., 2003).

The proteins ABI1, MPRIP, NDRG1, and JAG2, which are involved in a protein complex with TNIK, are identified as potential downstream substrates of TNIK at S707. ABI1_S231, MPRIP_T542, NDRG1_T366, and JAG2_S1208 showed positive expression co-regulation with TNIK_S707. In our analysis, CARMIL1, an actin cytoskeleton–associated protein, was identified as a potential downstream substrate of TNIK, which was recently reported as a potential upstream kinase phosphorylating CARMIL1 at S707 (Sheela et al., 2025). Furthermore, PAK4_S148 and CTNND1_T177 are positively co-regulated with S769 and identified as potential downstream substrates for TNIK.

### 3.9 Association of co-phosphoregulated proteins of TNIK phosphosites with Wnt signaling

Given that TNIK promotes cell proliferation in multiple cancers, such as colorectal cancer, ovarian cancer, and lung squamous cell carcinoma, through the Wnt signaling pathway, we further assessed the co-phosphoregulation patterns of Wnt pathway genes with the predominant sites of TNIK (Supplementary Figure S1). DVL3_S48, CSNK2B_S209, SIRT1_S47, and CHD8_S2048 showed positive expression co-regulation with TNIK_S640. Among them, SIRT1 is the binary interactor of TNIK, and the phosphorylation of SIRT1 at S47 increases its nuclear localisation and enzymatic activity (Nasrin et al., 2009). SIRT1 regulates the Wnt signaling pathway by interacting with DVL proteins, and can affect the Wnt target gene expression (Lin and Fang, 2013). With TNIK_S680, FZD6_S620, SENP2_S333, the sites S2093, S1042 and S2088 of APC, NFATC4_S289, TCF7L2_S60, FOSL1_S74 showed positive co-regulation. Among them, SENP2 and FZD6 are reported to form a protein complex with TNIK. SENP2 and APC are regulators of the Wnt/β-catenin signaling pathway (Bu et al., 2018), (Benchabane and Ahmed, 2009). FZD6 is a Wnt receptor that regulates the canonical Wnt pathway (Dong et al., 2023). CSNK2B_S205, PRICKLE2_S753, TLE4_S292, TLE4_S292, GSK3A_S21, and sites S9 and S13 of GSK3B, a binary interactor of TNIK, showed positive expression co-regulation with TNIK_S707. GSK3B is a negative regulator of Wnt signaling, where its phosphorylation at S9 inhibits its enzymatic activity (Li et al., 2017; Treekitkarnmongkol et al., 2024). Additionally, when GSK3B is phosphorylated at S9, its interaction with CTNNB1 is disrupted and blocked (Chen et al., 2020). With TNIK_S769, EP300_S1038, FBXW11_S65, SIRT1_S47, NFATC4_S289, PSEN1_S367, CHD8_S1424, TLE1_S263, CAMK2D_T287 showed positive expression co-regulation. Likewise, the phosphorylation of CAMK2D at T287 is associated with its induced enzymatic activity (Simon et al., 2021). TLE3_S267 is negatively co-regulated with TNIK_S707, and CAMK2D_T331 is negatively co-regulated with S769 of TNIK.

### 3.10 Comparative analysis of TNIK_S764 with the predominant site TNIK_S769

**FIGURE 4:**
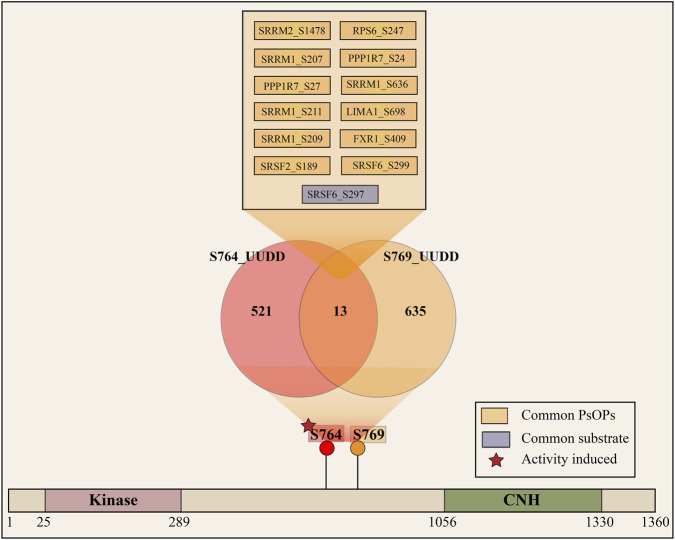
Venn diagram showing the overlap of positively co-regulated phosphosites (PsOPs) associated with TNIK_S764 and the predominant site TNIK_S769. Only 13 PsOPs were shared, with SRSF6_S297 as the sole common downstream site.

S764 is the only known phosphosite predicted to be an activity-inducing site of TNIK. It has been reported to be associated with increased cell growth and prostate cancer (Lee et al., 2019). Since S764 is located near the predominant site S769, we performed a comparative analysis of both phosphosites to investigate any potential functional overlap. Our analysis revealed that S764 functions independently of the predominant sites, as only 13 PsOPs were found to be positively co-regulated with both S764 and S769 (Figure 4). Among them, only one downstream site is shared, which is SRSF6_S297. The number of potential downstream substrates co-regulating with S764 is lower compared to our predominant sites, suggesting that the predominant sites could be exerting a larger influence on the kinase activity. Additionally, a kinase-dead mutant study of TNIK conducted by Jiang et al. (2024) (Jiang et al., 2024) revealed that TNIK_S769 is a kinase activity-associated site.

## 4 Discussion

Among the 35 phosphosites reported in the differential data, we analyzed the prominent phosphosites that are frequently perturbed in TNIK (S640, S680, S707, and S769). Evolutionary conservation analysis indicated TNIK S640 a highly conserved phosphosite, while both S707 and S769 were identified to be moderately conserved, and S680, poorly conserved. To determine whether the predominant sites of TNIK are exposed to the protein surface and accessible to solvents, we performed RSA and PAI analysis. The results revealed that the predominant sites S640, S707 and S769, exhibited structural accessibility with the highest score obtained for S769, while S680 is slightly less exposed. However, the high PAI score of S680 indicates the preserved accessibility in a flexible structural context. This suggests that S769 resides in a highly flexible and exposed segment of the protein, consistent with regulatory phosphorylation.

TNIK plays multifunctional roles in cancer cell proliferation, treatment resistance, and cell migration across cancer types and is largely dependent on its activation of Wnt signaling (Ewald et al., 2024). It is reported to interact with both β-catenin and TCF4 and phosphorylates TCF4 to promote TCF/LEF-dependent transcription of Wnt target genes (Masuda et al., 2015). Our analysis of co-phosphoregulated proteins with TNIK_S640 phosphorylation revealed a set of proteins strongly associated with these functions. Among these, PEA15_S116, SMC4_S28, HTT_S1874, NMD3_S468, PNKP_S114, and CBX8_S256 showed the highest positive co-regulation, and are known to play key roles in Wnt signaling (Su et al., 2025; Ramakrishna et al., 2023), (Zhu et al., 2024; Vergara et al., 2017), (Lee et al., 2012; Segal-Raz et al., 2011). Importantly, the phosphorylation of the top co-regulated protein PEA15 at S116 enhances the expression of β-catenin and its translocation to the nucleus. With TNIK_S680, TRIM24_S1025 is one of the top positive co-phosphoregulated proteins, and APC_S2093, APC_S1042, TCF7L2_S60, and FOSL1_S74 associated with wnt signaling were also found co-regulated. Wnt/β-catenin signaling is required for the function of TRIM24 in CRC cells (Tian et al., 2022). Similarly, ABI1_S231 and LMNA_S66 shown to have positive expression co-regulation with TNIK_S707 are involved in Wnt signaling (Veltrop et al., 2024), (Nath et al., 2019). With TNIK_S769, SIRT1_S47, LRRFIP2_S328, and TRIM24_S1028 are positively co-phosphoregulated and are associated with Wnt signaling (Lin and Fang, 2013; Tian et al., 2022; Liu et al., 2005). Furthermore, our analysis identified phosphorylation sites in proteins positively co-regulated with the predominant TNIK phosphosites (listed in Supplementary Table S5) associated with carcinogenesis, cell growth, and inhibition of apoptosis. The phosphorylation at DPYSL3_T509 (Braekeveldt et al., 2018), PTPN11_Y542 (Karaca et al., 2022), RANBP1_S60 (Huynh et al., 2019), SIRT1_S47 (Yao et al., 2022), and SLC9A1_S703 (Amith et al., 2016), which are associated with these functions, is positively co-regulated with S640 of TNIK. Similarly, phosphorylation at MVD_S96 (Larson-Casey et al., 2014), IGF2BP1_S181 (Lambrianidou et al., 2021), USP11_S948 (Li et al., 2022), PAK4_S291 (Huang et al., 2022), OSTF1_S202 (Tanimura et al., 2011), PTK2_Y576 (Sinnett-Smith et al., 2022) and KAT7_T88 (Duong et al., 2013) is positively co-regulating with S680 of TNIK. Furthermore, BRAF_S446 (Park et al., 2022), BAZ1B_S158, (Liu et al., 2020), IWS1_S720 (Sanidas et al., 2014), and MAP2K4_S80 (Park et al., 2002) showed positive expression co-regulation with TNIK_S707 and phosphorylation at YAP1_S138 (Zhao et al., 2014), SIRT1_S47 (Yao et al., 2022), ILF3_S382 (Ding et al., 2020), SHCBP1_S273 (Shi et al., 2021) and TP53BP2_S698 (Xiao et al., 2023) with TNIK_S769.

**FIGURE 5:**
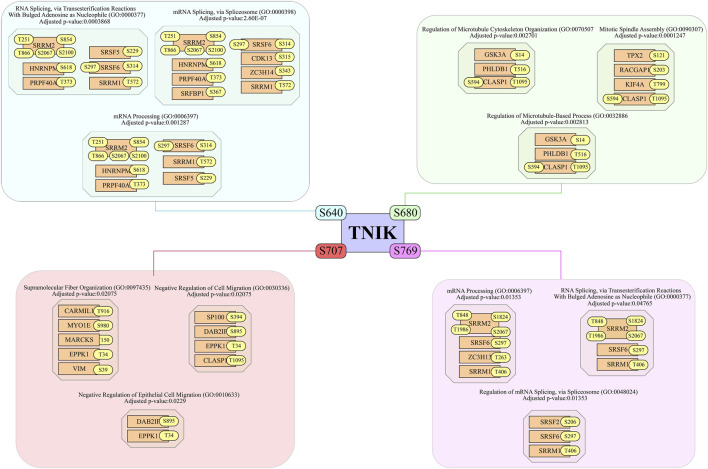
The diagram illustrates the co-regulatory network of TNIK phosphorylation sites S640, S680, S707, and S769 with downstream substrates. Each arm of the schematic figure represents a specific phosphosite and its associated proteins, categorized based on functional roles.

Recently, it has been reported that TNIK knockdown can affect the splicing of XBP1 under ER stress and reduce apoptosis (Samanthapudi et al., 2025). In line with this, our analysis revealed the potential positively co-phosphoregulated downstream substrates of TNIK, with its predominant sites S640 and S690, which are involved in the splicing (Figure 5). The different phosphorylation sites of potential substrates SRRM1, SRSF6, and SRRM2 showed co-regulation with both S640 and S769. SRSF6 is a potential oncogenic gene that promotes oncogenic splicing (She et al., 2021). SRSF6_S297 is positively co-regulating with both S640 and S769. The phosphorylation at T572 of SRRM1, an important factor in the alternative splicing (Song and Ma, 2022), is positively co-regulated with TNIK_S640, while SRRM2_T1986 and T848 positively co-regulate with S769 of TNIK, and T866, T251, and S2100 co-regulate with TNIK_S640, while SRRM2_S2067 is co-regulated with both S640 and S769 of TNIK. SRRM2 promotes interactions between messenger RNA and the spliceosome catalytic machinery (Cuinat et al., 2022). The phosphorylation of TNIK at S640, S680, and S769 is reported to be increased in SARS-CoV-2 infection (Huuskonen et al., 2024; Undi et al., 2023). The potential s ubstrates of TNIK corresponding to their predominant sites, S640 and S769 align their phosphorylation mechanisms to RNA splicing. Likewise, the predicted substrates (Supplementary Table S7), including KIF4A, CLIP1, MINK1, and VIM, are positively co-phosphoregulated with site S680 of TNIK, associated with cytoskeletal organization. KIF4A, a chromokinesin positively co-regulated with TNIK_S680, shows increased maximal microtubule-dependent ATPase activity associated with its T99 phosphorylation site (Nunes Bastos et al., 2013). CLIP1 binds to the plus end of microtubules and promotes microtubule growth and affects cytoskeletal organization (Guharoy et al., 2013), and its S204 site is positively co-regulated with TNIK_S680. The site S763 of MINK1, a member of the germinal centre kinases known to regulate cytoskeletal organization, is positively co-regulated with S680 of TNIK. S51 of VIM, which is associated with cytoskeletal reorganization, is positively co-regulated with S680 (Goto et al., 2002). Similarly, for site S707 of TNIK, the potential downstream substrates, including DAB2IP_S895 (Yang et al., 2011), CLASP1_T1095 (Kumar et al., 2009), IRS2_S577 (Gorgisen et al., 2022), and EPPK1_T34 (Kokado et al., 2013), which are associated with epithelial cell migration, showed positive expression co-regulation. A kinase mutant study of TNIK suggests its role in the disruption of F-actin structure and regulation of the cytoskeleton (Fu et al., 1999), also the kinase activity is required for activation of Wnt target genes (Mahmoudi et al., 2009). Additionally, the positively co-regulated cytoskeleton associated proteins CLIP2_S294 and CAMSAP2_S1313 were found to be downregulated in the kinase dead mutant study of TNIK conducted by Jiang et al. (2024) (Jiang et al., 2024).

A knockdown study in *Drosophila* revealed that TNIK could be a regulator of DHAP-mTORC1 signaling (Pham et al., 2023). mTORC1 coordinates cell growth according to extracellular and intracellular conditions. Active mTORC1 promotes the synthesis of proteins, lipids, and nucleotides for cell growth by phosphorylating multiple downstream targets, including p70 ribosome protein S6 kinase (S6K1), eukaryotic translation initiation factor 4E-binding protein (4E-BP1), and autophagy-associated kinase Unc-51-like kinase 1 (ULK1) (Zhu and Wang, 2020). Even though no studies have reported the association of TNIK with mTOR signaling, our analysis revealed substrates of mTOR signaling to be positively co-regulated with predominant sites of TNIK, which include potential upstream kinases and substrates. EIF4EBP1 is a potential substrate of TNIK. EIF4EBP1_T70 is positively co-regulated with S640 and S680 of TNIK, which is also a downstream target of mTORC1 during cell growth (Zhu and Wang, 2020). RPTOR, a predicted downstream substrate of TNIK, plays an essential role in the subcellular localisation of mTORC1 and also regulates its assembly and recruits kinase substrates such as EIF4EBP1 (Sengupta et al., 2010). RPTOR_T865 is positively co-regulated with S680 and S769. PRKAA1, which is a negative regulator of mTOR signaling and also identified as the potential upstream kinase of TNIK, is positively co-regulated with predominant sites of TNIK. PRKAA1_S496 is co-regulated with S640 of TNIK, while S486 of PRKAA1 is co-regulated with S707. The phosphorylation of PRKAA1 at S496 inhibits its AMPK activity (Heathcote et al., 2016), which can lead to an increased anabolic process. RPS6KB2, which is a downstream target of mTORC1 (Khalil et al., 2024), is identified as a potential upstream kinase of TNIK. RPS6KB2_S417 is positively co-regulated with S640 of TNIK, while RAF1, is identified as the predicted kinase of TNIK, can activate MEK/ERK, which can enhance mTORC1 activity through modulation of TSC2 and other intermediates (Saini et al., 2013). RAF1_S301 is positively co-regulated with S680 of TNIK.

Our study suggests the importance of TNIK in controlling important physiological functions by means of site-specific phosphorylation events, connecting it to growth signaling pathways, cytoskeletal organization, mRNA splicing, and epithelial migration. Our findings broaden the regulatory role of TNIK in alternative splicing and possible crosstalk with mTORC1 signaling, in addition to its well-established role in Wnt signaling and cytoskeletal regulation. The co-regulatory patterns seen between TNIK phosphosites and splicing regulators such as SRRM1, SRSF6, and SRRM2 highlight the recently identified role of TNIK in RNA processing. Furthermore, the overlap with apoptotic and growth-promoting substrates and the phosphosite-specific co-regulation with mTOR signaling components suggests a broader role for TNIK in oncogenic signaling and cellular homeostasis.

## 5 Conclusion

Our study based on the global co-phosphoregulation analysis highlights the central role of TNIK as a multifunctional kinase involved in cancer progression, RNA splicing, cytoskeletal reorganization, and its potential connection with the mTOR signaling pathway. The phosphorylation of TNIK at sites S640, S680, S707, and S769 was found to be strongly associated with the regulation of critical cellular processes, including cell proliferation, inhibition of apoptosis, and epithelial migration. Importantly, these predominant sites of TNIK shows positive co-phosphoregulation with proteins including upstream kinases and downstream substrates, many of which are implicated in apoptosis, cytoskeletal dynamics, and mTOR-mediated anabolic processes. Our analysis supports TNIK’s candidature as a promising therapeutic target in cancer and other diseases marked by aberrant signaling through Wnt, cytoskeletal, splicing, and mTOR-related pathways. Our analysis offers a systems-level view of TNIK signaling, enabling the identification of context-specific functional associations across diverse cellular processes. This approach not only uncovers novel regulatory axes of TNIK but also provides a valuable framework for future studies aimed at dissecting its role in disease-specific signaling networks.

## Data Availability

The original contributions presented in the study are included in the article/Supplementary Material, further inquiries can be directed to the corresponding author.
