# Supplementary material for: A system-wide investigation into the phosphoregulatory network of TNIK and its cellular implications
Source: Front Bioinform. 2026 Mar 13;6:1722876. doi: 10.3389/fbinf.2026.1722876 (PMC13021626; doi:10.3389/fbinf.2026.1722876)
Supplement: Supplementary file 3 [file Supplementaryfile1.docx]

**Title: A System-wide Investigation into the Phosphoregulatory Network of TNIK and its Cellular Implications**


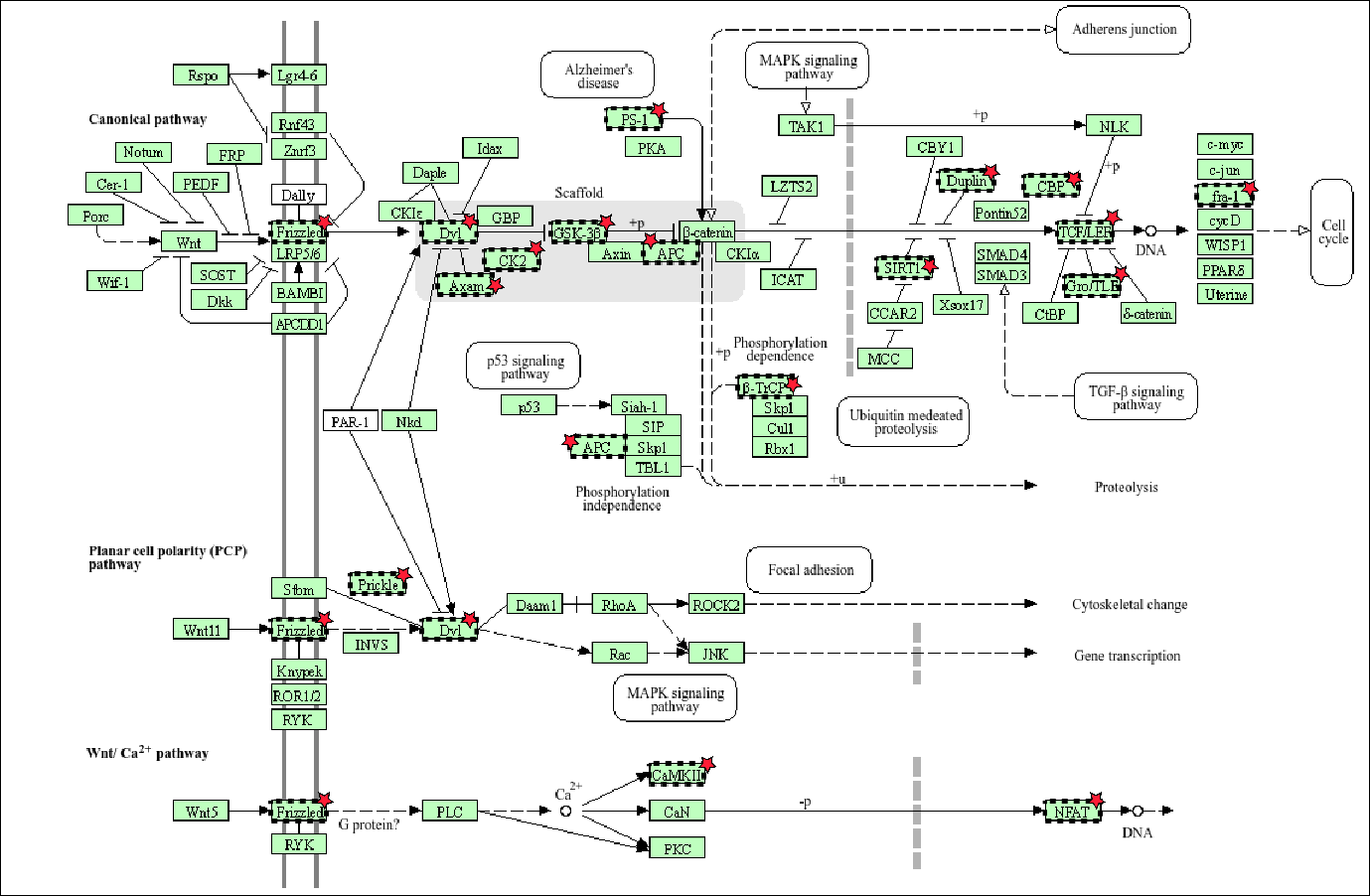


**Supplementary Figure 1.** Proteins involved in the Wnt signaling pathway that show phosphosite coregulations with TNIK.
